# Combined predictive values of neutrophil-lymphocyte ratio and platelet-lymphocyte ratio for surgical site infection after emergency cesarean section: a retrospective case-control study

**DOI:** 10.3389/fsurg.2025.1670583

**Published:** 2025-09-16

**Authors:** Xuemei Yang, Weina Yang, Qianqian He, Xiuqing Zhou, Qianwen Liu, Haiying Li

**Affiliations:** ^1^ Department of Obstetrics, Shijiazhuang Obstetrics and Gynecology Hospital, Shijiazhuang, Hebei, China; ^2^ Department of Neonatal Surgery, Hebei Children’s Hospital, Shijiazhuang, Hebei, China

**Keywords:** emergency cesarean section, surgical site infection, neutrophil-to-lymphocyte, platelet-to-lymphocyte, predictive value

## Abstract

**Objective:**

This study aims to evaluate the predictive value of neutrophil-to-lymphocyte ratio (NLR) and platelet-to-lymphocyte ratio (PLR) independently and in combination for surgical site infection (SSI) after emergency cesarean section (CS).

**Method:**

This retrospective case-control study was conducted at the Maternity Medical Centre in China, a large tertiary teaching medical centre, between January 2019 and February 2022. A total of 627 patients with emergency CS were enrolled, and cases (post-SSI) and controls (without post-SSI) were matched 1:2. Various demographic, clinical and obstetric characteristics were collected. Laboratory values were measured on preoperative and postoperative days 1, 3. Univariate and multivariate logistic regression analyses were conducted to identify the influencing factors. The predictive values of NLR and PLR independently and in combination for SSI were evaluated using receiver operating characteristics (ROC) analysis.

**Result:**

In the univariate analysis, the BMI before delivery, preoperative NLR and PLR, and postoperative day 1 NLR and PLR et al. were significantly different between the two groups (*P* < 0.05). In multivariate analysis, BMI before delivery > 28.89 kg/m^2^, preoperative NLR > 9.89 and PLR > 177.99 appeared to be independent predictors of SSI after emergency CS. Combined indices of BMI before delivery, NLR and PLR were significantly more predictive of SSI after emergency CS than independent indices (AUC 0.85, *P* < 0.001, 95% CI 0.757–0.912, sensitivity 79.1%, and specificity 88.5%).

**Conlusion:**

The combined index of BMI before delivery, preoperative NLR and PLR may be a simple, sensitive, inexpensive, versatile, and rapid test for predicting SSI after emergency CS. Of course, further prospective research and external verification will be more scientific and also our future work focus.

## 1 Introduction

Cesarean section (CS) is one of the most common obstetric procedures worldwide, and the number of CS has increased over the past decade (1). Compared with vaginal delivery, CS is associated with a 5–20 times higher risk of postpartum infections, ranging from endometritis to urinary tract and wound infections (2). Surgical site infection (SSI) following CS is a common complication and a major cause of morbidity and mortality, affecting the recovery process and increasing both hospitalisation and medical costs (3, 4). The incidence of SSI after CS varies between 3% and 30% (5, 6). For this reason, it is important to predict and prevent the development of SSI after CS as early as possible. Several observational studies have reported that emergent CS, multiple gestation, volume of blood loss, operative technique, duration of surgery, and maternal obesity are common risk factors for SSI (7–10). The risk of SSI after emergency CS was approximately 20% higher than that after elective surgery (7).

It is know that neutrophils, monocytes, and platelets have important roles in normal wound healing. There has been increasing evidence that the hematological indicators neutrophil-to-lymphocyte ratio (NLR) and platelet-to-lymphocyte ratio (PLR) may be used as markers of systemic inflammation, reflecting inflammatory status and activity in a variety of diseases such as infectious diseases, metabolic syndrome, lung diseases, malignant tumours and cardiovascular diseases (11–15). Furthermore, some other studies have preliminarily shown that NLR and PLR may play an increasingly important role in obstetrics and may be effective markers for predicting postpartum infection, early abortion, threatened abortion, pre-eclampsia and hyperemesis gravidarum (11, 12, 16–18).

In recently studies, the role of NLR and PLR in predicting SSI after CS was examined, and NLR was found as a potential marker (19). Additionally, the PLR has been reported to be an independent predictor of reduced survival and has a negative prognostic value in gynaecological diseases and hepatobiliary malignancies (20). Previous studies have tentatively confirmed the independent predictive value of NLR and PLR after CS infection (16). However, the independent predictive value and combined predictive value of NLR and PLR for SSI after emergency CS remains unclear. Therefore, the aim of this study was to evaluate the predictive value of NLR and PLR in SSI after emergency CS, independently and in combination, to aid in their early prediction.

## 2 Materials and methods

### 2.1 Study design and participants

This retrospective case-control study was conducted at the Shijiazhuang Obstetrics and Gynecology Hospital in Hebei, China, between January 2019 and February 2022. Our hospital is one of the largest maternity hospitals in Hebei Province with approximately 30,000 deliveries per year. This study was approved by the Institutional Review Board of Shijiazhuang Obstetrics and Gynecology Hospital (approval number 20230040). Informed consent was waived by the same ethics committee that approved the study.

Six hundred and twenty-seven pregnant women who underwent emergency CS and were followed up at our institution at one month with a maternal postnatal examination were considered eligible for the study. Cases were defined as patients undergoing emergency CS who experienced an SSI within 30 days of the procedure; controls were patients undergoing emergency CS who did not experience an SSI within 30 days of the procedure. Cases (with SSI) were matched to controls (without SSI) on a 1:2 basis.

Inclusion criteria were as follows: (1) adult patients aged ≥ 22y; (2) patients who underwent energency CS between January 2019 and February 2022; (3) patients in the case group with a positive wound swab indicating infection within 30 days of surgery; (4) patients in the control group without a postive wound swab; (5) patients in the control and case groups were of similar age and received CS on the same day. The following groups were excluded from the study: (1) patients undergoing elective CS; (2) patients with chronic systemic diseases that may alter the preoperative complete blood count, such as systemic lupus erythematosus, nephropathy, renal or hepatic dysfunction, rheumatoid arthritis; (3) pregnancies with known chromosomal abnormalities, congenital malformations, eclampsia, or hypertensive disorders; (4) patients with missing data, known maternal infection, recent use of corticosteroids, various hematological conditions, tuberculosis or malignant tumour disease; (5) patients who died either before or on the same day as the blood test were excluded from the analysis.

SSI was defined as an infection occurring within 30 days of a postoperative procedure involving the skin, subcutaneous tissue, soft tissue, or any other part of the body (21). The diagnostic criteria for SSI were in assordance with relevant guidelines (22): (1) patients with body temperature higher than 38℃; (2) the percentage of neutrophils was higher than 70%, and the white blood cell count was less than 4.0 × 10^9^/L or higher than 10.0 × 10^9^/L; (3) the pathogenic bacteria were cultured in incision secretions.

### 2.2 Data collection of variables

Data were extracted from Hospital In-Patient Enquiry database (HIPE) for the period 2019–2022. The HIPE database is a computerized system designed to capture the administrative, demographic and clinical data on all inpatient discharges in the Shijiazhuang Obstetrics and Gynecology Hospital. The medical records including maternal age, place of residence, body mass index (BMI) before delivery, parity, previous cesareans section, gestational weeks, diabetes, operative time, type of anesthesia, and blood loss. BMI was calculated from pre-pregnancy weight and height. In addition, complete blood count (CBC) results routinely obtained on day 1 and 3 postoperatively were included. The following blood count variables were analyzed: hemoglobin (HGB), red blood cells (RBC), white blood cells (WBC), albumin (ALB), C-reactive protein (CRP), neutrophils, lymphocytes, platelets. The NLR and PLR were calculated by dividing the total neutrophil count and the total platelet count by the total lymphocyte count, respectively.

It is worth noting that all blood samples were collected by drawing 5 ml of blood from the antecubital vein without the use of anticoagulants by professionally trained phlebotomists. To control for circadian rhythms, all samples were routinely collected during the same time period (6:00 am to 8:00 am) on the preoperative day and on days 1 and 3 postoperatively. The samples were transported to the biochemistry laboratory within one hour for testing, which was performed in our hospital's hematology laboratory using the same Beckman Coulter Gen-S automated analyzer (Brea, CA, United States) for all samples.

### 2.3 Surgical procedures

All emergency CS were performed by consultants and senior obstetric residents. At our institution, prophylactic antibiotics are routinely administered for emergency CS. Prophylactic antibiotics were administered with a single dose of 1 g cefazoline 30–60 min before starting the skin incision. As standard for all emergency CSs, the anesthetist administers general or local anesthesia, depending on the patient. During surgery, both the abdomen and vagina were sterilized with 10% povidone-iodine disinfectant immediately after spinal anaesthesia. After delivery, the abdominal fascia was closed with 0 monofilament continuous sutures. After the incision sites were washed with saline, a 4–0 monofilament dermostitch suture was performed. There were no reported cases of subcutaneous drains among the samples analyzed. After surgery, the incision sites were covered with sterile dressings, which were removed on postoperative day 3.

### 2.4 Statistical analysis

Data were analyzed using SPSS 21.0 (IBM, Armonk, NY). Continuous variables with a normal distribution are presented as mean ± SD (standard deviation). The Shapiro–Wilk test was used to assess the normality of continuous variables. For nonnormally distributed continuouus variables, the Mann–Whitney *U*-test was used. Categorical variables are presented as counts and percentages, using chi-squared or Fisher's exact test when appropriate. Besides, univariate and multivariate logisitcs regression analyses was utilized to identify identify influencing factors for SSI after emergency CS. Variables with significant differences (*P* < 0.05) were entered into a multiple logistic regression model to assess whether there was an independent association between hematological indices and ISS of emergency CS. In all cases, a significance threshold of *P* < 0.05 was used.

## 3 Results

### 3.1 Patient characteristics

During a 3-year study, a total of 627 pregnant women who underwent emergency CS surgery were included in the study (mean age 27.6 ± 3.2 years). There were 209 patients diagnosed with postoperative SSI (group 1) and 418 patients without postoperative SSI (group 2). The BMI before delivery, operative time and preoperative and postoperative day 1 and 3 neutrophil count, lymphocyte count, PLT count, NLR, PLR, CRP were determined by ROC curve analysis (Table 1).

**Table 1:** Optimal cut-off value of contiuous variables detected by ROC curve analysis.

Variables	Cut-off value	Area under the ROC curve (AUC)	95% CI	*P*-value
BMI before delivery (kg/m^2^)	28.89	0.71	0.54–0.80	0.066
Preoperative Neutrophil count (10^−9^/L)	10.30	0.61	0.50–0.72	0.066
Preoperative Lymphocyte count (10^−9^/L)	1.30	0.63	0.52–0.74	0.026
Preoperative PLT count (10^−9^/L)	212.15	0.59	0.48–0.71	0.119
Preoperative NLR	9.89	0.72	0.61–0.82	<0.001
Preoperative PLR	177.99	0.66	0.55–0.77	0.007
Preoperative CRP	35.39	0.43	0.31–0.55	0.245
Operative time (min)	42.50	0.68	0.58–0.79	0.001
Postoperative 1st day Neutrophil count (10^−9^/L)	9.99	0.59	0.48–0.70	0.114
Postoperative 1st day Lymphocyte count (10^−9^/L)	0.76	0.58	0.46–0.69	0.173
Postoperative 1st day PLT count (10^−9^/L)	283.75	0.51	0.39–0.62	0.92
Postoperative 1st day NLR	11.98	0.62	0.51–0.73	0.046
Postoperative 1st day PLR	243.33	0.57	0.45–0.68	0.267
Postoperative 1st day CRP	78.31	0.54	0.42–0.65	0.554
Postoperative 3rd day Neutrophil count (10^−9^/L)	7.74	0.59	0.48–0.70	0.114
Postoperative 3rd day Lymphocyte count (10^−9^/L)	0.71	0.58	0.47–0.79	0.173
Postoperative 3rd day PLT count (10^−9^/L)	345.42	0.51	0.39–0.62	0.920
Postoperative 3rd day NLR	7.68	0.53	0.41–0.64	0.646
Postoperative 3rd day PLR	187.83	0.45	0.34–0.56	0.400
Postoperative 3rd day CRP	81.35	0.51	0.39–0.62	0.892

*P* value < 0.05 was considered statistically significant.

ROC, receiver operating characteristic; AUC, areas under the curve; CI, confidence interval; NLR, neutrophil-to-lymphocyte ratio; PLR, platelet-to-lymphocyte ratio; CRP, C-reactive protein; PLT, platelet.

In the univariate analysis, the BMI before delivery, parity, preoperative neutrophil count > 10.30 × 10^−9^/L, lymphocyte count < 1.30 × 10^−9^/L, PLT count > 212.15 × 10^−9^/L, NLR > 9.89 × 10^−9^/L, PLR > 177.99 × 10^−9^/L, CR*P* > 35.39 mg/L, operative time > 42.50 min, intraoperative blood loss > 400 ml, 1st day postoperative neutrophil count > 9.99 × 10^−9^/L, HGB < 115 g/L, NLR > 11.98 (*P* < 0.001), and 1st day postoperative PLR >243.33 (*P* = 0.002) of patients in group 1 were significantly different from those in group 2 (Table 2). The multivariate logistic regression analysis model included the significant associated factors shown in Table 1. The final multivariate analysis results indicated that preoperative NLR > 9.89 (OR 4.39, 95% CI 1.79–12.57, *P* = 0.001), preoperative PLR > 177.99 (OR 3.55, 95% CI 0.81–15.53, *P* = 0.033), BMI before delivery > 28.89 kg/m^2^ (OR 8.65, 95% CI 3.24–14.36, *P* = 0.048) were independent risk factors for SSI after emergency CS (Table 3).

**Table 2:** Univariate analyses of variables associated with SSI after emergency CS.

Variable	Post SSI (*N* = 209)	Without post SSI (*N* = 418)	*P* value
Maternal age (years, mean ± SD)	27.56 ± 3.31	27.54 ± 3.83	0.983
BMI before delivery (>28.89 kg/m^2^, cutoff), *n* (%)	179 (85.65)	146 (34.93)	**<0**.**001**
Parity (≥1), *n* (%)	81 (38.76)	242 (57.89)	**<0**.**001**
Previous CS (yes), *n* (%)	34 (16.27)	85 (20.33)	0.221
Gestational diabetes (yes), *n* (%)	8 (3.82)	30 (7.17)	0.098
Gestational hypertension (yes), *n* (%)	31 (14.83)	74 (17.70)	0.364
Smoking during pregnancy (yes), *n* (%)	9 (4.31)	26 (6.22)	0.325
Gestational age (weeks, mean ± SD)	37.21 ± 3.63	38.12 ± 5.00	0.582
Preoperative hematologic indicators			
Neutrophil count (>10.30 × 10^−9^/L, cutoff), *n* (%)	174 (83.25)	242 (57.89)	**<0**.**001**
Lymphocyte count (<1.30 × 10^−9^/L, cutoff), *n* (%)	145 (69.38)	169 (40.43)	**<0**.**001**
PLT count (>212.15 × 10^−9^/L, cutoff), *n* (%)	131 (62.68)	154 (36.84)	**<0**.**001**
WBC (>10 × 10^−9^/L, reference), *n* (%)	199 (95.21)	389 (93.06)	0.293
RBC (<3.5 × 10^−12^/L, reference), *n* (%)	63 (30.14)	132 (31.58)	0.714
HGB (<115 g/L, reference), *n* (%)	185 (88.52)	367 (87.80)	0.794
ALB (<40 g/L, reference), *n* (%)	83 (39.71)	190 (45.45)	0.172
CRP (>35.39 mg/L, cutoff), *n* (%)	39 (18.66)	22 (5.26)	**<0**.**001**
NLR (>9.89 cutoff), *n* (%)	127 (60.77)	88 (21.05)	**<0**.**001**
PLR (>177.99, cutoff) *n* (%)	107 (51.20)	103 (24.64)	**<0**.**001**
Intraoperative indexes			
Anesthetization, *n* (%)			0.528
General	18 (8.61)	39 (9.33)	
Intraspinal	191 (91.39)	179 (90.67)	
Operative time (minutes), *n* (%)			**<0**.**001**
0–42.50 cutoff	107 (51.20)	332 (79.43)	
>42.50	102 (48.80)	96 (20.57)	
Intraoperative blood loss (ml), *n* (%)			**<0**.**001**
0–400 reference	87 (41.63)	83 (19.86)	
>400	122 (58.37)	335 (80.14)	
Blood transfusion (yes), *n* (%)	9 (4.31)	16 (3.83)	0.773
The duration of urinary catheter (h), *n* (%)			0.673
0–24 reference	139 (66.51)	285 (68.18)	
>24	70 (33.49)	133 (31.82)	
Hematologic indexes of the postoperative 1st day			
Neutrophil count (>9.99 × 10^−9^/L, cutoff), *n* (%)	184 (88.04)	265 (63.40)	**<0**.**001**
Lymphocyte count (<0.76 × 10^−9^/L, cutoff), *n* (%)	87 (41.63)	164 (39.23)	0.564
PLT count (>283.75 × 10^−9^/L, cutoff), *n* (%)	87 (41.63)	181 (43.30)	0.689
WBC (>10 × 10^−9^/L, reference), *n* (%)	145 (69.38)	264 (63.16)	0.123
RBC (<3.5 × 10^−12^/L, reference), *n* (%)	140 (66.99)	250 (59.81)	0.081
HGB (<115 g/L, reference), *n* (%)	175 (83.73)	288 (68.90)	**<0**.**001**
ALB (<40 g/L, reference), *n* (%)	97 (46.41)	175 (41.87)	0.279
CRP (>78.31 mg/L, cutoff), *n* (%)	53 (25.36)	117 (27.99)	0.485
NLR (>11.98, cutoff), *n* (%)	93 (44.50)	104 (24.88)	**<0**.**001**
PLR (>243.33, cutoff) *n* (%)	102 (48.80)	149 (35.65)	**0**.**002**
Hematologic indexes of the postoperative 3rd day			
Neutrophil count (>7.74 × 10^−9^/L, cutoff), n(%)	141 (67.46)	253 (60.53)	0.090
Lymphocyte count (<0.71 × 10^−9^/L, cutoff), *n* (%)	29 (13.88)	80 (19.14)	0.101
PLT count (>345.4 × 10^−9^/L,cutoff), *n* (%)	53 (25.36)	102 (24.40)	0.793
WBC (>10 × 10^−9^/L, reference), *n* (%)	126 (60.29)	256 (61.24)	0.817
RBC (<3.5 × 10^−12^/L, reference), *n* (%)	145 (69.38)	271 (64.83)	0.256
HGB (<115 g/L, reference), *n* (%)	169 (80.86)	322 (77.03)	0.273
ALB (<40 g/L, reference), *n* (%)	102 (48.80)	178 (42.58)	0.140
CRP (>81.35 mg/L, cutoff), *n* (%)	83 (39.71)	146 (34.93)	0.464
NLR (>7.68, cutoff), *n* (%)	68 (32.54)	163 (39.00)	0.114
PLR (>187.8, cutoff) *n* (%)	178 (85.17)	338 (80.86)	0.183

*P* value < 0.05 were marked in bold, indicating a statistically significant difference.

BMI, body mass index; PLT, platelet; NLR, neutrophil-to-lymphocyte ratio; PLR, platelet-to-lymphocyte ratio; CRP, C-reactive protein; WBC, white blood cell; RBC, red blood cell; HGB, hemoglobin.

**Table 3:** Mutivariable logistic regression analyses of variables associated with SSI after emergency CS.

Variable	Multivaruate analysis		*P* value
	OR	95% CI	
BMI before delivery (kg/m^2^)	8.65	3.24–14.36	**0**.**048**
Parity (≥1), *n* (%)	0.65	0.51–0.84	0.154
Preoperative hematologic indicators			
Neutrophil count (>10.30 × 10^−9^/L, cutoff), *n* (%)	0.92	0.85–1.01	0.204
Lymphocyte count (<1.30 × 10^−9^/L, cutoff), *n* (%)	0.16	0.02–1.31	0.481
PLT count (>212.15 × 10^−9^/L, cutoff), *n* (%)	1.08	0.97–1.21	0.068
CRP (>35.39 mg/L, cutoff), *n* (%)	1.38	0.57–2.33	0.179
NLR (>9.89 cutoff), *n* (%)	4.39	1.79–12.57	**0**.**001**
PLR (>177.99, cutoff) *n* (%)	3.55	0.81–15.53	**0**.**033**
Intraoperative indexes			
Operative time (>42.5 min), *n* (%)	1.11	0.66–1.21	0.207
Intraoperative blood loss (>400 ml), *n* (%)	0.70	0.53–0.93	0.170
Hematologic indexes of the postoperative 1st day			
Neutrophil count (>9.99 × 10^−9^/L, cutoff), *n* (%)	2.87	0.57–14.35	0.012
HGB (<115 g/L, reference), *n* (%)	0.59	0.32–1.24	0.341
NLR (>11.98, cutoff), *n* (%)	0.89	0.29–1.06	0.846
PLR (>243.33, cutoff) *n* (%)	0.95	0.64–1.13	0.211

*P* value < 0.05 were marked in bold, indicating a statistically significant difference.

BMI, body mass index; PLT, platelet; NLR, neutrophil-to-lymphocyte ratio; PLR, platelet-to-lymphocyte ratio; CRP, C-reactive protein; WBC, white blood cell; RBC, red blood cell; HGB, hemoglobin; OR, odds ratio; CI, confidence interval.

### 3.2 Indepentent indices

**Figure 1:**
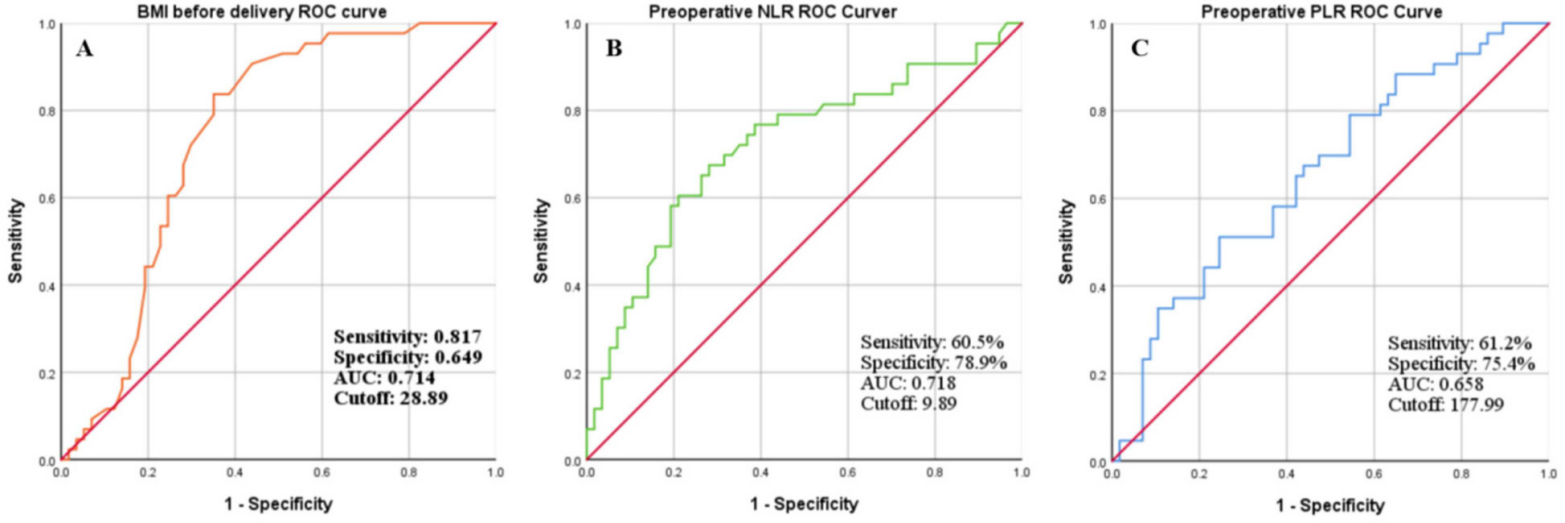
ROC curve analysis was performed to determine the cut-off value of **(A)** BMI before delivery; **(B)** preoperative NLR; and **(C)** preoperative PLR and to calculate the sensitivity, specificity and AUC for independently predicting SSI after emergency CS.

In this study, BMI before delivery > 28.89 kg/m^2^, peroperative NLR > 9.89 and PLR > 177.99 were significantly increased in the postoperative SSI group compared to the no SSI group (*P* < 0.001). ROC curve analysis was performed independently for the prediction of SSI in emergency CS. For BMI before delivery > 28.89 kg/m^2^, AUC was 0.714, sensitivity was 0.817, and specificity was 0.649%. For peroperative NLR > 9.89, AUC was 0.718, sensitivity was 0.605 and specificity was 0.789. For PLR > 177.99, AUC was 0.658, sensitivity was 0.612 and specificity was 0.754 (Figure 1).

### 3.3 Combined indices

**Figure 2:**
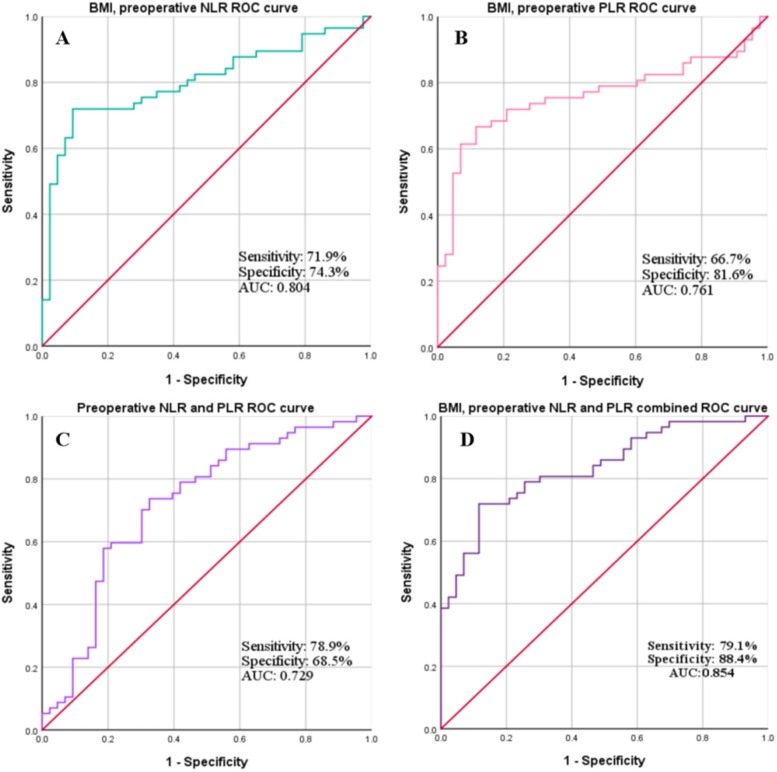
ROC curve analysis was performed to calculate the sensitivity, specificity and AUC for predicting SSI after emergency CS using a combination of 2 or more indics **(A)**, BMI before delivery combined with preoperative NLR; **(B)**, BMI before delivery combined with preoperative PLR; **(C)**, preoperative NLR combined with preoperative PLR; **(D)**, BMI before delivery combined with preoperative NLR and PLR.

According to the combined predictive probabilities by logistic regression analysis, ROC curves were used to evaluate the predictive values of the combination of BMI before delivery > 28.89 kg/m^2^, preoperative NLR > 9.89 and PLR > 177.99 in the SSI group compared with with no SSI. For BMI before delivery > 28.89 kg/m^2^ combined with preoperative NLR > 9.89, the AUC was 0.804, the sensitivity was 0.719 and the specificity was 0.743. For BMI before delivery > 28.89 kg/m^2^ combined with preoperative PLR > 177.99, AUC was 0.761, sensitivity was 0.667, specificity was 0.816. For preoperative NLR > 9.89 combined with PLR > 177.99, AUC was 0.729, sensitivity was 0.789, specificity was 0.605. For BMI before delivery > 28.89 kg/m^2^ combined with preoperative NLR > 9.89 and PLR > 177.99, the AUC was 0.854, sensitivity was 0.791, specificity was 88.47, better than individually independent or pariwise combined index of BMI before delivery, preoperative NLR and PLR (Figure 2).

## 4 Discussion

In recent years, many studies have found that NLR and PLR may be associated with the further development of infection after CS (16, 23). To our knowledge, this is the first study to use the simple, inexpensive laboratory indicators in combination to predict postoperative SSI in patients with emergency CS. In our study, the results of multiple logistic regression analysis showed that preoperative NLR > 9.89 (OR 4.39, 95% CI 1.79–12.57, *P* = 0.001), preoperative PLR > 177.99 (OR 3.55, 95% CI 0.81–15.53, *P* = 0.033), BMI before delivery > 28.89 kg/m^2^ (OR 8.65, 95% CI 3.24–14.36, *P* = 0.048) were independent predictors of SSI after emergency CS. In addition, the combined predictive value of preoperative BMI, NLR and PLR (AUC was 0.854, sensitivity was 0.791, specificity was 0.884) was significantly better than that of independent or pariwise combined indices.

Emergency CS is a common procedure worldwide, and with increasing rates of CS, the occurrence of SSI following emergency CS is a major clinical and public health concern (7, 9). Much international literature has identified several risk factors that predispose an individual to develop SSI following CS in general, including obesity and increased BMI, increased age, existing comorbidities, prolonged operative time, intraoperative blood loss and the complexity of an emergency CS (8, 24, 25). In our study, BMI > 28.89 kg/m^2^ is a significant risk factor for developing SSI after emergency CS, which is consistent with previous research demonstrating a negative impact on the risk of postoperative infection (24). Previous studies have shown that impaired immune response, larger wound area and poor perfusion of prophylactic antibiotics in obese individuals may account for this increased risk. Another possible explanation is that BMI plays a role in emergency CS due to the potential dysfunction that excess adipose tissue can cause to the immune system and a decrease in periorperative tissue oxygenation (26). Therefore, increased monitoring and control of antenatal BMI in this group may effectively mitigate the potential development of postoperative SSI.

Especially in the past 20 years, the relationship between these hematologic markers and many diseases has been examined. SSI is a clinical condition in which healing is impaired as a result of an infection, blood cells will have an important effect on the development of SSI, both in terms of inflammation and wound healing (27). However, the number of studies examining the relationship between preoperative and postoperative inflammatory hematologic markers and SSI is limited. Some studies have shown that NLR and PLR were the most commonly used inflammatory markers among CBC parameters and were reliable markers of systemic inflammation and may reflect both pro- and anti-inflammatory states (12, 15). These markers have been studied as novel predictors of several diseases, including cardiovascular disease, sepsis, irritable bowel syndrome, inflammatory bowel disease, rheumatic diseases, pulmonary diseases, malignancy, and others (12, 28, 29). In obstetrics, some scholars still have dispute over the predictive value of NLR and PLR for SSI after CS. Some scholars believe that NLR and PLR may not be effective and useful parameters to predict SSI after CS (30). However, a larger number of scholars believe that NLR and PLR are independent markers of postpartum infection, pre-eclampsia, preterm labour in pregnant women and many other gynecological conditions (18, 23). Rotem et al. (16). showed that both NLR and PLR during the first 24 postoperative hours may have a predictive value in the early detection of post-CS infection. However, the number of studies on the combination of NLR and PLR to predict SSI after emergency CS is limited.

SSI is one of the most common infections in CS, which is mostly superficial and usually occurs 4–7 days after surgery (29). Early prediction of SSI after emergency CS is a major challenge to control the disease and improve surgical outcomes. Currently, no combination screening test for the prediction of postoperative SSI has gained widespread acceptance in clinical practice. It is of utmost importance to find simple predictive parameters before the development of classic clinical signs and symptoms of SSI after emergency CS (31, 32). To reduce the rate of postoperative SSI, our medical center has adopted routine measures such as perioperative antibiotic use, chlorhexidine skin preparation, same level of surgery, same surgical strategy and no sutures. In our study, multivariate logistic regression analysis showed that preoperative NLR > 9.89, preoperative PLR > 177.99 and BMI > 28.89 kg/m^2^ were independent risk factors for SSI after emergency CS. However, ROC curve analysis shows that the predictive value of the three indices independently (BMI, AUC was 0.714, sensitivity was 0.817 and specificity was 0.649; NLR, AUC was 0.718, sensitivity was 0.605 and specificity was 0.789; PLR, AUC was 0.658, sensitivity 0.612, specificity was 0.754) and pairwise combination (BMI combined with NLR, AUC was 0.804, sensitivity was 0.719 and specificity was 0.743; BMI combined with PLR, AUC was 0.761, sensitivity was 0.667, specificity was 0.816; NLR combined with PLR, AUC was 0.729, sensitivity was 0.789, specificity was 0.605) is significantly lower than the predictive value of the three indices combined (BMI combined with NLR and PLR, AUC was 0.854, sensitivity was 0.791, specificity was 0.885). Therefore, we believe that our findings have clinical implications; patients with BMI before delivery > 28.89 kg/m^2^, preoperative NLR > 9.89 and preoperative PLR > 177.99 may benefit from closer monitoring. Early detection and treatment of postoperative infections may also have economic implications.

Our study has several limitations. First, this is a retrospective and single-center study, which has its inherent flaws. Second, this study could not investigate the detailed pathogenesis of elevated NLR and PLR levels in emergency CS patients with SSI. Additionally, other laboratory test results of certain clinical and inflammatory markers, such as interleukin (IL)-6, IL-10, erythrocyte sedimentation rate, and TNF-α, were not included in the analysis because they were not measured in all patients. Finally, due to the short hospital stay of CS patients in our center, only whole blood samples were taken before surgery and 1, 3 days after surgery were used for analysis. Therefore, the results of this study should be verified in further prospective and multicenter studies.

## 5 Conclusions

In conclusion, the results of the present study have demonstrated that patients with emergency CS with BMI > 28.89 kg/m^2^, NLR > 9.89 and PLR > 177.99 should receive targeted intervention and close monitoring to prevent SSI. The combined index of BMI, preoperative NLR and PLR is a simple, sensitive, inexpensive, versatile and rapid test for predicting SSI in patients undergoing emergency CS. Controlling BMI, reducing preoperative NLR and PLR is beneficial for the prevention of SSI. In addition, further studies are warranted to elucidate the risk factors for SSI after CS to provide evidence for the management of CS.

## Data Availability

The original contributions presented in the study are included in the article/Supplementary Material, further inquiries can be directed to the corresponding author.
